# Complete Genome Sequence of *Tea Plant Necrotic Ring Blotch Virus* Detected from a Tea Plant in Japan

**DOI:** 10.1128/mra.00323-22

**Published:** 2022-05-18

**Authors:** Noriko Maruyama, Nozomu Iwabuchi, Masanobu Nishikawa, Takamichi Nijo, Tetsuya Yoshida, Yugo Kitazawa, Kensaku Maejima, Shigetou Namba, Yasuyuki Yamaji

**Affiliations:** a Department of Agricultural and Environmental Biology, Graduate School of Agricultural and Life Sciences, The University of Tokyo, Tokyo, Japan; DOE Joint Genome Institute

## Abstract

We report the complete genome sequence of a Japanese isolate of *Tea plant necrotic ring blotch virus* (TPNRBV-J). The predicted TPNRBV-J genes have the same organization as those of a Chinese isolate, and the 5′ termini of the segments have conserved nucleotide sequences.

## ANNOUNCEMENT

*Tea plant necrotic ring blotch virus* (TPNRBV) is a member of the genus *Blunervirus*, in the family *Kitaviridae* (1, 2). TPNRBV has four positive-sense single-stranded RNA genome segments, designated RNA1 to -4 (1). TPNRBV was identified in China and Iran from tea plants (Camellia sinensis [L.] O. Kuntze) with discoloration and necrotic ring blotches on the leaves (1, 3). To date, the complete nucleotide sequence has been determined for only one TPNRBV isolate from China (1). In Japan, while several diseases with virus-like symptoms have been recorded in tea plants (4–6), there have been no reports on the detection of TPNRBV or its sequences.

In 2018, leaves of a tea plant showing necrotic ring blotches were collected in the Kanto region of Japan. Total RNA was extracted from leaves using the ISOSPIN plant RNA kit (Nippon Gene, Japan). A partial fragment each from TPNRBV RNA1 to -4 was amplified by reverse transcription-PCR (RT-PCR) using TPNRBV-specific primer pairs (1), cloned into pCR Blunt II-TOPO vector (Invitrogen, USA), and sequenced using Sanger sequencing. A BLASTn search (7) showed that each fragment had the highest nucleotide sequence identities (90 to 97%) with the Chinese TPNRBV isolate (1). To determine the complete genome sequence of the Japanese isolate (TPNRBV-J), nine overlapping fragments excluding the 5′ and 3′ ends were amplified by RT-PCR using primers designed from the Chinese isolate (Table 1). The 5′ and 3′ terminal regions of each genome segment were amplified using a GeneRacer kit (Invitrogen) with TPNRBV-specific or oligodeoxythymidylic acid [oligo(dT)] primers (Table 1). Each fragment was cloned and sequenced as described above. The complete genome sequence of each segment was constructed by assembling all overlapping fragments using ATGC v. 4.3.5 (Genetyx, Japan) with default settings, which showed 100% identity in the overlapping regions.

**TABLE 1:** List of primers used in this study

Primer	Sequence (5′ to 3′)	Position^*a*^	Purpose^*c*^
GeneRacer oligo(dT) primer	GCTGTCAACGATACGCTACGTAACGGCATGACAGTGTTTTTTTTTTTTTTTTTT	poly (A)	Reverse transcription
TPNRBV1-F^*b*^	GCCCTGACAACGCAAAAGAACTGATG	296–321	Detection of RNA1
TPNRBV1-R^*b*^	GTGACGGGATATTTTTGGACGACTGT	830–805	Detection of RNA1
TPNRBV2-F^*b*^	GGGCCGGGGTGTGGAAAAACTT	1022–1043	Detection of RNA2
TPNRBV2-R^*b*^	TTCTTATCATCCCGGCAAAACACA	1572–1549	Detection and PCR for RNA2 (nt 37–1572)
TPNRBV3-F^*b*^	TTCGCCACTCACAAAGACAACAAACT	1542–1567	Detection of RNA3
TPNRBV3-R^*b*^	GTAGCGGAGCGGAAAGAAAAGACT	2000–1977	Detection of RNA3
TPNRBV4-F^*b*^	TCAGTGGCGCGATTATCAGAAGGTA	1709–1733	Detection of RNA4
TPNRBV4-R^*b*^	CGCGCAAGAAGTCGGTCAAAAC	1955–1933	Detection of RNA4
TPNRBV1-35F	GCACAGAATACCATATTTTAAAC	35–57	PCR for RNA1 (nt 35–1664)
TPNRBV1-1664R	GAATTTATCCGCCTCACTTTA	1664–1644	PCR for RNA1 (nt 35–1664)
TPNRBV1-1560F	GCTGGTTGTCGCGATCTATA	1560–1579	PCR for RNA1 (nt 1560–3285)
TPNRBV1-3285R	AGACCCAACATACTGATCGAT	3285–3265	PCR for RNA1 (nt 1560–3285)
TPNRBV1-3181F	CATTTTGATCATTTGAAAAGGA	3181–3202	PCR for RNA1 (nt 3181–4872)
TPNRBV1-4872R	AACAACATCTATAGAAGATATCC	4872–4850	PCR for RNA1 (nt 3181–4872)
TPNRBV1-4767F	TGTCGAATCAGCGAAATCTA	4767–4786	PCR for RNA1 (nt 4767–5889)
TPNRBV1-5889R	ATAACGACGACGAAATAACTG	5889–5869	PCR for RNA1 (nt 4767–5889)
TPNRBV2-37F	CTGAAATCACCATATTTTAAACC	37–59	PCR for RNA2 (nt 37–1572)
TPNRBV2-1498F	GTCGTCGTATCCTTTGTCTAAG	1498–1519	PCR for RNA2 (nt 1498–3101)
TPNRBV2-3101R	CAAGTAACATGCATTCGATTAAC	3101–3079	PCR for RNA2 (nt 1498–3101)
TPNRBV2-3008F	GAAGCCGAGAACTATAACTGTG	3008–3029	PCR for RNA2 (nt 3008–4023)
TPNRBV2-4023R	CAATATAATGACAGTATCGTCG	4023–4002	PCR for RNA2 (nt 3008–4023)
TPNRBV3-68F	GTCGTTCTCTGGTCTAATAC	68–87	PCR for RNA3 (nt 68–2664)
TPNRBV3-1384R	ACAGGGTTGTTCTTTGCATAC	1384–1364	Sequencing for RNA3
TPNRBV3-1259F	TATGATGGTTTTACCATTCTGAC	1259–1281	Sequencing for RNA3
TPNRBV3-2664R	ACATACCACATGAAATGATTTG	2664–2643	PCR for RNA3 (nt 68–2664)
TPNRBV4-34F	GGTTGTTAATCACACCATATC	34–54	PCR for RNA4 (nt 34–2212)
TPNRBV4-830F	CCATCACACTCTATACCTAATATG	830–853	Sequencing for RNA4
TPNRBV4-2212R	ACATACCAATTGAGAAAATTTG	2212–2191	PCR for RNA4 (nt 34–2212)
TPNRBV1-204R	CGGAGGACTAAAACCGAGATTAT	204–182	5′ RACE for RNA1
TPNRBV1-5569F	AATGACATAAACCTTAACTACCC	5569–5591	3′ RACE for RNA1
TPNRBV2-221R	CGAGTTCCACTAACGATTTAGGTG	221–198	5′ RACE for RNA2
TPNRBV2-3848F	ACCGAGTTATGGTATATAAACC	3848–3869	3′ RACE for RNA2
TPNRBV3-113R	CATGTCAGATTGTTAGGTGGTAGCG	113–89	5′ RACE for RNA3
TPNRBV3-2555F	CTGAATAATAATATCGTTCGTAA	2555–2577	3′ RACE for RNA3
TPNRBV4-158R	GCCACGATAGCCAACTCAAGTGT	158–136	5′ RACE for RNA4
TPNRBV4-1929F	AAAAGTTTTGACCGACTTCTT	1929–1949	3′ RACE for RNA4
T7F	TAATACGACTCACTATAGGG	pCR-Blunt II-TOPO vector	Sequencing for TOPO vector
SP6	CATACGATTTAGGTGACACTATAG	pCR-Blunt II-TOPO vector	Sequencing for TOPO vector
M13F	GTAAAACGACGGCCAGT	pCR-Blunt II-TOPO vector	Sequencing for TOPO vector
M13R	CAGGAAACAGCTATGAC	pCR-Blunt II-TOPO vector	Sequencing for TOPO vector

a Numbers are based on TPNRBV-J (GenBank accession numbers LC566237 to LC566240).

b Hao et al. (1).

c RACE, rapid amplification of cDNA ends.

The complete RNA1 to -4 sequences excluding poly(A) tails at the 3′ ends were 5,912 (42.2% GC content), 4,118 (41.4% GC content), 2,684 (42.0% GC content), and 2,232 (44.7% GC content) nucleotides (nt) long, respectively. The National Center for Biotechnology Information (NCBI) open reading frame (ORF) finder (https://www.ncbi.nlm.nih.gov/orffinder/) was used to predict each ORF using default parameters. Putative protein functions were manually annotated using the NCBI conserved domain database (v. 3.19) using default parameters (8). RNA1 possessed a single ORF (nt 130 to 5715), which contained conserved methyltransferase, cysteine-protease, and helicase (HEL) domains. RNA2 possessed a single ORF (nt 134 to 3766), which contained conserved HEL domains and an RNA-dependent RNA polymerase. RNA3 possessed four ORFs encoding 14-kDa (nt 111 to 482), 29-kDa (nt 528 to 1280), 21-kDa (nt 1328 to 1876), and 22-kDa (nt 1929 to 2561) proteins with unknown functions. RNA4 possessed a single ORF (nt 398 to 1345), which contained a putative movement protein. A conserved nucleotide motif (5′-AATTACGA-3′) was found at the 5′ termini of RNA1 to -3, while RNA4 had a slightly different one (5′-ATTAACGA-3′). The sequence identity with the reported isolate was calculated using the MUSCLE algorithm (9) in the program SDT v. 1.2 (10). The amino acid sequence identities of each ORF compared to the Chinese isolate were 95.2 to 99.5%. Based on the proposed species demarcation criterion in the genus *Blunervirus*, which has less than 75% amino acid sequence identity for the single protein encoded by the RNA1 (11), TPNRBV-J was found to belong to the same species as the Chinese TPNRBV isolate.

### Data availability.

The genome sequence of TPNRBV-J has been deposited in the DDBJ under accession numbers LC566237 (RNA1), LC566238 (RNA2), LC566239 (RNA3), and LC566240 (RNA4).
